# Development and Implementation of a Smartphone Application to Promote Physical Activity and Reduce Screen-Time in Adolescent Boys

**DOI:** 10.3389/fpubh.2014.00042

**Published:** 2014-05-20

**Authors:** David R. Lubans, Jordan J. Smith, Geoff Skinner, Philip J. Morgan

**Affiliations:** ^1^Priority Research Centre for Physical Activity and Nutrition, School of Education, University of Newcastle, Callaghan, NSW, Australia; ^2^Faculty of Science and Information Technology, School of Design Communication and Information Technology, University of Newcastle, Callaghan, NSW, Australia

**Keywords:** physical activity, obesity prevention, sedentary behavior, behavior change, self-determination theory, social cognitive theory, technology, fitness and exercise

## Abstract

**Purpose:** To describe the development and implementation of a smartphone application (app) designed to promote physical activity and reduce screen-time in adolescent boys considered “at-risk” of obesity.

**Methods:** An app was developed to support the delivery of a face-to-face school-based obesity prevention program known as the “Active Teen Leaders Avoiding Screen-time” (ATLAS) program. ATLAS was guided by self-determination theory and social cognitive theory and evaluated using a cluster randomized controlled trial with 361 boys (12.7 ± 0.5 years) in 14 secondary schools. Following the completion of the study, participants in the intervention group completed a process evaluation questionnaire and focus groups were conducted with 42 students to explore their general perceptions of the ATLAS program and their experience with the smartphone app. Barriers and challenges encountered in the development, implementation, and evaluation of the app are also described.

**Results:** Participation in the study was not contingent on ownership of a smartphone, but 70% of participants in the intervention group reported having access to a smartphone or tablet device. Focus group participants reported an enjoyment of the program, and felt that it had provided them with new skills, techniques, and routines for the future. However, their engagement with the smartphone app was limited, due to a variety of reasons. Barriers to the implementation and evaluation of the app included limited access to smartphone devices, technical problems with the push notifications, lack of access to usage data, and the challenges of maintaining participants’ interest in using the app.

**Conclusion:** Although participants reported high levels of satisfaction with the ATLAS program in general, the smartphone app was not used extensively. Additional strategies and features may be needed to enhance engagement in adolescent boys.

## Introduction

Physical inactivity has been described as a global pandemic (1). Recent estimates suggest that approximately 80% of young people internationally are not meeting the physical activity guidelines of 60 min of moderate-to-vigorous physical activity (MVPA) each day (2). It is of additional concern that children and adolescents are spending a large proportion of their day engaged in screen-based recreation. Both physical inactivity and high levels of screen-time are associated with a range of adverse physical and psychological health outcomes in young people, including obesity, metabolic syndrome, and poor mental health (3–5). Although adolescent boys are typically more active than girls (2, 6), they report significantly higher levels of screen-time (7), making them susceptible to unhealthy weight gain and poor social and emotional well-being.

Schools have been identified as ideal settings for physical activity promotion and obesity prevention, as they have access to the majority of youth, appropriate facilities, and qualified personnel to achieve these outcomes (8). Numerous school-based interventions delivered in the primary-school setting with children have been found to be effective in promoting physical activity and preventing obesity (9, 10). In comparison, the evidence for effective school-based interventions targeting adolescents in secondary schools is limited. Indeed, the most recent Cochrane review of obesity prevention interventions found that primary school-based interventions were twice as successful as interventions targeting adolescents (9). The challenges of achieving health behavior change in this cohort has prompted researchers to explore novel and engaging intervention strategies. One such approach has involved the use of eHealth technology (e.g., internet, mobile phones, etc.) to encourage young people to develop physical activity behavioral skills (i.e., self-monitoring and goal setting) (11–13) and prevent the decline in physical activity typically observed during adolescence (14).

Mobile phone ownership is increasing at a rapid rate and recent data suggest that 77% of US adolescents (15) and 90% of Australian adolescents over the age of 15 own mobile phones (16). Not surprisingly, there has been a proliferation of mobile phone-based interventions using apps and short messaging service (SMS) to prompt physical activity and healthy eating in adults (17, 18). The evidence suggests that SMS-delivered interventions can have positive short-term behavioral outcomes in adults, but little is known regarding their utility for increasing activity levels in adolescents. A recent systematic review of smartphone apps for pediatric obesity prevention (19) found that very few apps included features recommended by the Expert Committee for Pediatric Obesity. The authors suggested that future apps should include comprehensive information about health behavior change and opportunities for goal setting. Although interventions are beginning to emerge in the published literature (20), little is known regarding the efficacy and practicality of mobile phone apps to promote physical activity and reduce sedentary behavior in young people.

Therefore, the primary objective of this paper is to describe the development and implementation of a smartphone app designed to support the delivery of the Active Teen Leaders Avoiding Screen-time (ATLAS) obesity prevention program (21). A secondary objective is to explore participants’ perceptions of the program in general. ATLAS was a multi-component school-based intervention targeting adolescent boys attending schools in low-income communities, who were considered to be “at-risk” of obesity based on their physical activity and screen-time behaviors.

## Materials and Methods

### Study design

Ethics approval for this study was obtained from the University of Newcastle, Australia and the New South Wales (NSW) Department of Education and Communities. School principals, teachers, parents, and study participants all provided informed written consent. The rationale, study protocol and intervention description have been reported previously (21). Briefly, ATLAS was evaluated using a cluster randomized controlled trial (RCT) conducted in state-funded co-educational secondary schools within low-income communities of NSW, Australia. The socio-economic indexes for areas (SEIFA) of relative socioeconomic disadvantage (scale 1 = *lowest* to 10 = *highest*) was used to identify eligible schools. The SEIFA index is derived from multiple indicators of socioeconomic disadvantage within an area (e.g., education, employment, etc.). Public secondary schools located in the Newcastle, Hunter, and Central Coast regions of NSW with a SEIFA index of ≤5 (lowest 50%) and an enrollment of at least 100 students in the targeted year group were considered eligible. Twenty-two eligible secondary schools were identified and 14 agreed to participate.

### Participants

A power calculation was conducted to determine the required sample size for detecting changes in the primary outcomes [i.e., Body Mass Index (BMI) and waist circumference]. Assuming a drop-out rate of 20% by the primary endpoint, it was calculated that 350 participants (i.e., 25 from each school) would be required to detect a between-group difference in BMI of 0.4 kg m^−2^ and 1.5 cm in waist circumference. All male students in the targeted year group at the study schools completed a short screening questionnaire to assess their eligibility for inclusion in the study. The questionnaire aimed to identify those “at-risk” of obesity based on their physical activity and screen-time behaviors. Based on their responses, students failing to meet national physical activity or sedentary behavior guidelines (22) were considered eligible and invited to participate. Students with a medical condition that would preclude them participating in the program were also excluded. In total, 361 adolescent boys (mean age, 12.7 ± 0.5 years) in Grade 7 (first year of secondary school) consented and completed baseline assessments.

### Intervention

Active Teen Leaders Avoiding Screen-time was informed by the Physical Activity Leaders (PALs) pilot study, a successful trial conducted in four secondary schools in the Hunter region, NSW, Australia (23–25). A detailed description of the ATLAS intervention is reported elsewhere (21). The multi-component intervention was designed to increase physical activity, reduce screen-time, and reduce intake of sugar-sweetened beverages among adolescent boys attending schools in low-income areas. The intervention, which was delivered over 20 weeks (February–June, 2013) and was underpinned by self-determination theory (SDT) (26) and social cognitive theory (SCT) (27). ATLAS focused on the promotion of lifetime (e.g., resistance training) and lifestyle (e.g., active transport) physical activities and was aligned with current physical activity guidelines, which include a recommendation to engage in muscle and bone strengthening physical activities on at least 3 days/week (22, 28). The intervention promoted four behavioral messages: (i) *walk whenever you can*; (ii) *get some vigorous physical activity on most days*; (iii) *reduce your recreational screen-time*; and (iv) *drink more water and less sugary drinks*. Briefly, the school-based intervention involved the following components: teacher professional development, researcher-led seminars, enhanced school sport sessions, lunch-time physical activity mentoring sessions, provision of fitness equipment to schools, pedometers for self-monitoring, parental strategies to reduce screen-time, and a smartphone application (app) and website. To assist in facilitating the intervention as intended, participating teachers at the study schools attended two full-day professional development workshops (pre- and mid-program) designed and delivered by the research team. While the other intervention components have been described previously (21), a detailed description of the app is provided below:

#### Smartphone application and website

**Figure 1:**
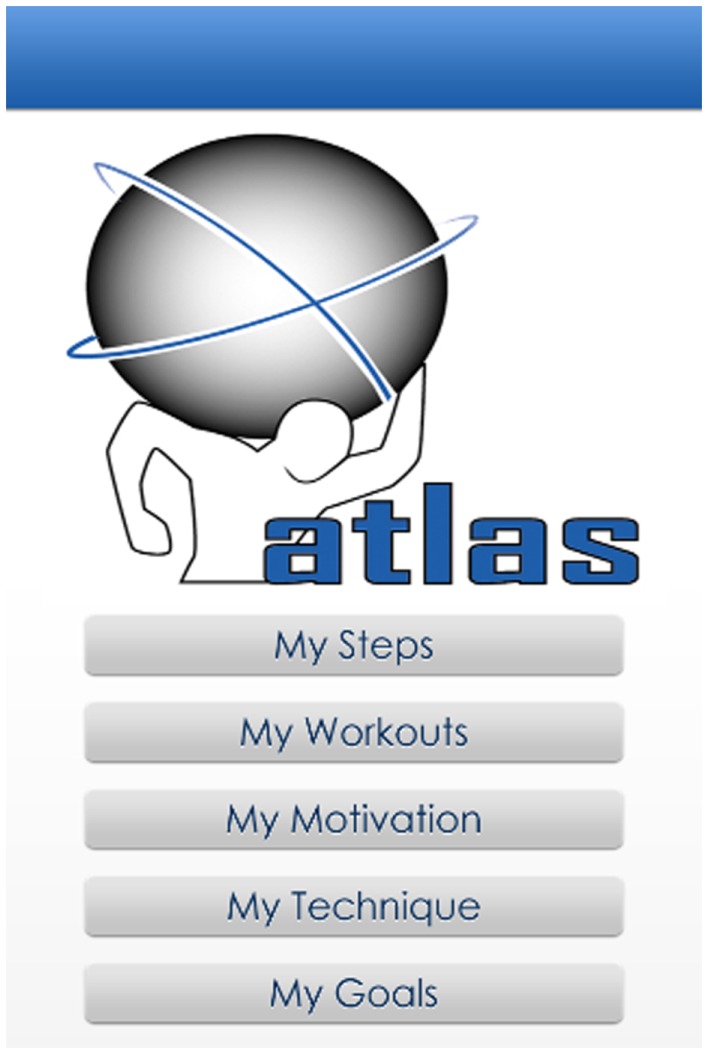
**ATLAS app welcome screen**.

A smartphone app was developed as a supplement to the intervention and was made available to participants on both iOS (i.e., Apple app store) and Android (i.e., Google Play) platforms at no cost to participants (Figure 1). A website was also developed so that the same features were available to participants without access to a smartphone or handheld device with similar capabilities. Data for both apps (i.e., iOS and Android) were stored on the device, but the iOS version could be backed up to a secondary location (i.e., iTunes or the Cloud). Consistent with the face-to-face components of the ATLAS intervention, the apps were operationalized using SDT (29) and SCT (27, 30) (Table 1). More specifically, the app was designed to satisfy participants’ needs for autonomy and to increase their autonomous motivation for physical activity. It was also designed to enhance their self-efficacy to be physically active and increase their outcome expectations regarding the benefits of physical activity and the consequences of excessive screen-time and sugared beverage consumption. Prompting of goal setting and behavioral monitoring were also encouraged. The five functions (Figure 1) of the app/website are described below.

**Table 1:** **ATLAS smartphone app features, behavior change techniques, and potential mediators**.

Feature	Description	Behavior change strategies	Potential mediators
Physical activity monitoring *(My steps)*	This feature enabled participants to record their daily step counts measured using a pedometer and review results over a daily, weekly, or monthly time scale	Prompt self-monitoring of behaviors	Autonomous motivation for physical activity
		Prompt specific goal setting	Behavioral capability
			Self-efficacy
			Self-control
Pre-designed fitness challenges *(My workouts)*	This feature listed 10 pre-designed CrossFit-style workouts including resistance training and aerobic exercises. Participants could enter and review results over a daily, weekly, or monthly time scale	Prompt self-monitoring of behaviors	Self-efficacy
		Set graded tasks	Self-control
			Behavioral capability
			Autonomous motivation for physical activity
			Autonomy support
Assessment of resistance training skill competency *(My technique)*	This feature enabled participants to assess their resistance training skill competency with the assistance of a peer or family member. Results could be reviewed over a daily, weekly, or monthly time scale	Prompt self-monitoring of behaviors	Behavioral capability
		Prompt practice	Self-efficacy
			Autonomous motivation for physical activity
Goal setting *(My goals)*	This feature allowed participants to set and review goals related to physical activity (steps/day), workouts (sessions/week), or screen-time (min/day). Push notifications were automatically sent to participants to confirm if goals were achieved	Prompt specific goal setting	Self-efficacy
		Prompt intention formation	Self-control
		Prompt self-monitoring of behaviors	Autonomous motivation for physical activity
			Motivation to limit screen-time
			Autonomy support
Tailored motivational messaging *(My motivation)*	Informational and motivational messages were sent twice weekly via push notifications through the app	Information on consequences	Outcome expectations
		Provide information about behavior–health link Provide general encouragement	Outcome expectancies Social support Autonomous motivation for physical activity Motivation to limit screen-time

##### Physical activity monitoring

*My steps* – participants were able to record their daily step counts measured using their personal pedometer which was provided by the research team. They could then review their “date-stamped” step count entries or select the graph view which allowed a visual representation (i.e., bar graph) of their entries over time. The graph view could be converted to show entries over a daily, weekly, or monthly time scale.

##### Pre-designed fitness challenges

**Figure 2:**
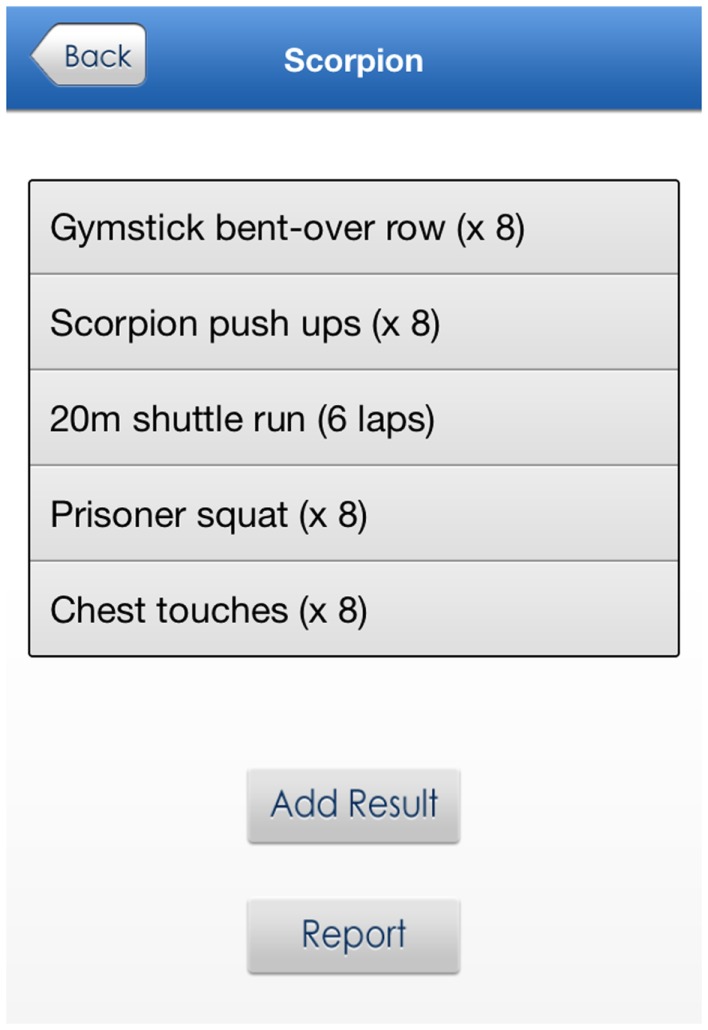
**Sample challenge workout**.

*My workouts* – during the school sport sessions participants were introduced to CrossFit-style fitness challenges (henceforth referred to as workouts), which involved a series of resistance training (e.g., push-ups) and aerobic exercises (e.g., shuttle runs) with a predetermined number of repetitions (see Figure 2 for example). The workouts were also included in the app to encourage participants to complete them outside of school hours. Ten separate workouts of “easy,” “moderate,” and “hard” rating were designed for the study and participants were encouraged to select workouts based on their perceived fitness levels. The time taken to complete the circuit was considered the result, with decreases in the time taken indicating improvements in performance. Using the app/website, students were able to select a workout, and then once completed, record their result (i.e., completion time). “Date-stamped” entries could be reviewed and entries could also be viewed in a bar graph format as described above.

##### Assessment of resistance training skill competency

*My technique* – the performance criteria for resistance training exercises from the Resistance Training Skills Battery (RTSB) (31, 32) were provided on the app/website. The RTSB is an assessment tool for appraising *technique* during the performance of six skills (i.e., squat, lunge, push-up, overhead press, suspended row, and front support with chest touches), which are considered to be the foundation for more complex movements used in resistance training programs (33–35). The app/website allowed users to assess their own (or others) technique, with the assistance of a peer or family member, during the performance of each resistance training skill. The performance criteria for each skill that were successfully demonstrated could be selected from a list of all criteria and then submitted following the completion of the exercise. The number of performance criteria successfully demonstrated is saved as the user’s score. “Date-stamped” entries could be reviewed and the graph view, using the format previously described, could also be used to track progress in correct performance of the resistance training skill over time.

##### Goal setting

*My goals* – the app/website allowed users to set and review goals related to physical activity and screen-time. This function enabled users to select either (a) the number of daily steps they would like to achieve; (b) the number of workouts per week they would like to complete; or (c) the amount of screen-time (in minutes) they would like to limit themselves to each day. The user could then select the date on which they would like the achievement of the goal to be reviewed. On the date selected by the user, a *push notification* was sent asking the user to verify achievement of the goal. Previously achieved goals were retained and displayed on the screen for user review.

##### Tailored motivational messaging

**Figure 3:**
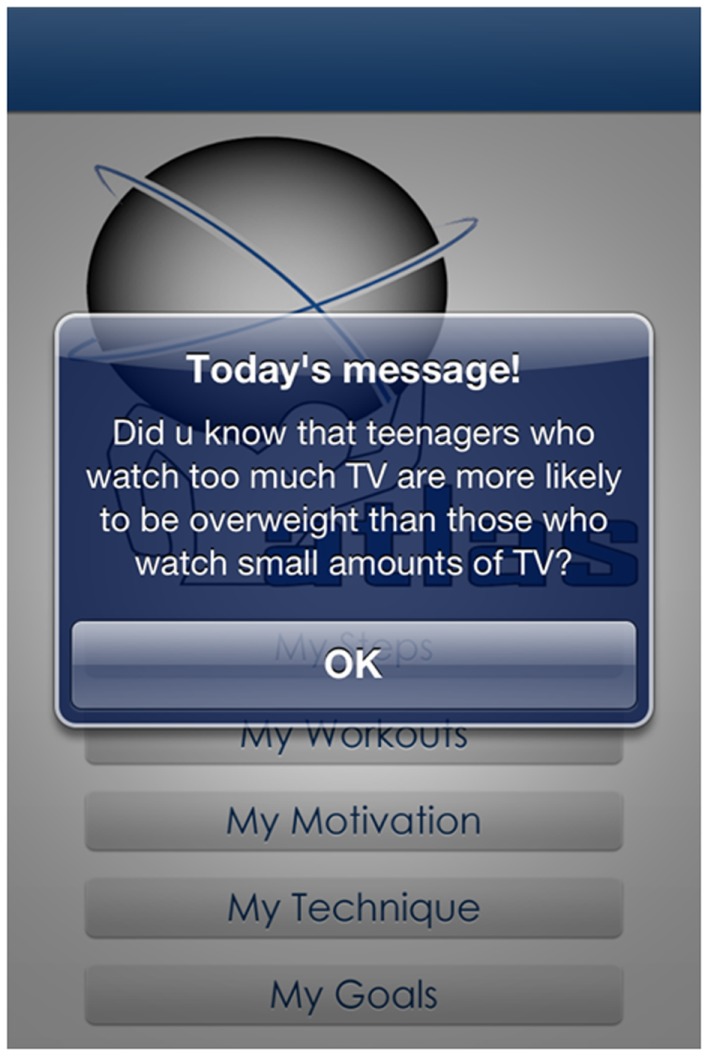
**Example-tailored motivational message**.

*My motivation* – this function was available on the app only. After the initial download of the app, users were asked to select two of four physical activity outcome expectations that were personally important to them, relating to (i) appearance (i.e., to look good), (ii) health and well-being (i.e., to improve my health), (iii) school performance (i.e., to do better at school), and (iv) social interaction (i.e., to spend time with friends). Based on their responses, informational and motivational messages developed in reference to SDT and SCT were sent twice weekly via *push notifications* through the app (see Figure 3 for example). The informational messages related to the ATLAS behavioral messages (e.g., *exercise helps u look fit and feel good. How much exercise have u done 2day?*) and the motivational messages were based on the user’s initial responses to the motivation question. (e.g., *Do u want to look good and feel gr8? Well u won’t get there sitting down!*). As recommended in the literature, messages were simple and written in vernacular “*text speak*” to engage teenagers (36).

### Process evaluation

Baseline and post-program assessments were conducted in November–December, 2012 and July–September, 2013, respectively. Trained research assistants completed data collection at the study schools. A process evaluation was conducted to determine participants’ usage of, and satisfaction with, the ATLAS app. Evaluation questionnaires were distributed to study participants at mid- and post-program periods. The mid-program questionnaire included items on the type of app/website usage (i.e., iOS, Android, or website usage) and the frequency of use (i.e., 1 = *Never* to 5 = *5 or more times*). The post-program questionnaire included more detailed items regarding user enjoyment of the app/website (1 = *Strongly disagree* to 5 = *Strongly agree*) and frequency of use for each specific function (1 = *Never* to 4 = *Often*).

Participants were also asked their behavioral intentions to (i) *limit recreational screen-time*, (ii) *limit consumption of sugary drinks*, (iii) *participate in at least 60 min of MVPA each day*, and (iv) *participate in muscle strengthening physical activities on 2–3 days each week* (1 = *Strongly disagree* to 5 = *Strongly agree*). To assist researchers interested in the use of smartphone apps for obesity prevention research, barriers and challenges encountered in the development, implementation, and evaluation of the app are described.

### Focus groups

A series of focus groups were conducted to gain insights into participants’ experiences in and perceptions of, the ATLAS program. Consenting students took part in separate focus groups, each consisting of six participants. Each focus group included three participants who failed to meet MVPA guidelines and three participants who achieved MVPA guidelines (using baseline accelerometer data). These group meetings lasted between 42 and 58 min and were conducted in a separate classroom during school hours by a research team member who had not been directly involved in the delivery of the ATLAS program. The structured discussion framework was developed by the research team to facilitate discussion and reflection on the program. Specifically, the questions asked of the students were designed to explore their general perceptions of ATLAS and their experience with the ATLAS smartphone app. Views were also sought of the participants as to the perceived impact of the program on a range of attitudes and behaviors relating to physical activity and nutrition. Prompts were used as needed to explore topics in depth.

#### Qualitative data analysis

The focus groups were digitally recorded with the participants’ consent and transcribed verbatim. A computer program (NVIVO 10) was used to assist with the organizational aspects of data analysis. Analysis was conducted by an independent qualitative researcher using a standard general inductive approach to qualitative analysis. Initially, inductively derived codes or labels were attached to the meaning units arising from the data. The developing hierarchical coding scheme was continually revised and further expanded after coding of additional transcripts. Following coding of all the transcripts, emerging themes were identified and elaborated. Due to the structured format of the discussion framework, these themes were closely aligned with the research aims. The following reports on these inductive analyses and explores the impact that the program has had on the lived experiences of the students taking part in the ATLAS program.

## Results

### Demographics

Participants were 361 adolescent males (mean age = 12.7 ± 0.5 years) attending schools in low-income areas of NSW, Australia. The majority of boys (i.e., 95%) were born in Australia and most (i.e., 96%) reported speaking English at home. Ninety two percent of boys reported their cultural background as Australian or European. Furthermore, 13.5% of boys indicated they were of Indigenous descent (i.e., Aboriginal or Torres Strait Islander). The sample was predominantly of low socioeconomic position with 91.4% of boys residing within areas with a SEIFA population decile ≤5 (i.e., bottom 50%). Twenty-nine percent of boys resided in areas with a SEIFA population decile ≤2 (i.e., bottom 20%).

### App/website usage

Participation in the study was not contingent on ownership of a smartphone, but 70% of participants in the intervention group reported having access to a smartphone or tablet device (including iPod Touch). At the mid-program evaluation, 49 and 15% of participants had used the iPhone and Android apps, respectively (Table 2). At the end of the intervention period, the majority of participants (70%) reported using the goal setting function to increase their physical activity or reduce their screen-time. Fewer participants used the app to monitor their resistance training technique (62%), pedometer steps (49%), and fitness challenge results (49%). Approximately, 20% of participants did not engage with the app at all.

**Table 2:** **Mid- and post-program process evaluation questions and responses**.

Questions	*N* (%)
**Mid-program questions**
I have used the iPhone app	53 (48.6)
I have used the Android app	14 (14.7)
I have used the website	25 (25.3)
Frequency of use	
≤2 times	70 (58.8)
≥3 times	49 (41.2)
**Post-program questions**
I enjoyed using the app/website
Strongly disagree	3 (2.8)
Disagree	13 (8.5)
Neutral	46 (43.4)
Agree	36 (34.0)
Strongly agree	12 (11.3)
The app messages reminded me to be more active, reduce my screen-time, and drink less sugary drinks	
Strongly disagree	6 (5.7)
Disagree	13 (12.4)
Neutral	35 (33.3)
Agree	38 (36.2)
Strongly agree	13 (12.4)
I used the “my goals” setting function on the app
Often	20 (19.0)
Sometimes	54 (51.4)
Rarely	15 (14.3)
Never	16 (15.2)
I used the “my technique” function on the app
Often	22 (20.8)
Sometimes	45 (42.5)
Rarely	21 (19.8)
Never	18 (17.0)
I used the “my steps” function on the app
Often	13 (12.4)
Sometimes	39 (37.1)
Rarely	26 (24.8)
Never	27 (25.7)
I used the “my workouts” function on the app
Often	16 (15.1)
Sometimes	37 (34.9)
Rarely	25 (23.6)
Never	28 (26.4)
How often did you wear your pedometer?
Often	34 (30.1)
Sometimes	50 (44.2)
Rarely	20 (17.7)
Never	9 (8.0)

*119 and 114 participants completed the mid- and post-program evaluations*.

### App/website satisfaction and behavioral intentions

After completing the program, almost half of the group agreed or strongly agreed that the push prompt messages reminded them to be more active, reduce their screen-time, and drink less sugary drink (Table 2). Forty-four percent of participants agreed or strongly agreed that the ATLAS app was enjoyable to use. Alternatively, 95% of participants agreed or strongly agreed that the ATLAS program overall was enjoyable. Participants’ intentions to limit their recreational screen-time (mean = 3.95 ± 1.07), limit their consumption of sugary drinks (mean = 4.01 ± 0.82), participate in regular MVPA (mean = 4.16 ± 0.81), and muscle strengthening activities (mean = 4.08 ± 0.76), were high following the completion of the program.

### Focus group results

A total of 42 male students from year 8 participated in 7 focus groups. Each group consisted of students attending the ATLAS program from the same school. The thematic analysis revealed a range of emerging themes surrounding the participants’ general perceptions of the program, key messages, and ATLAS app, as well as clusters relating to the students’ perceptions and evaluations of the physical activity sessions, as well as relationships with teachers and peers. The overarching theme relating to the perceived impact of the ATLAS program contained a number of sub-themes representing the changes to behaviors, knowledge, and attitudes relating to school, diet, and physical activity which were felt to have followed on directly as a result of involvement in the ATLAS program.

#### General perceptions of ATLAS

While a number of students had some suggestions for how the program could have been improved (mainly in terms of less repetitive activities and more variety), all expressed an enjoyment of the program, and felt that it had provided them with new skills, techniques, and routines for the future, while learning about the importance of reducing sedentary behavior, adopting a healthy diet, and limiting sugary drinks also generally having been received well by the students;

“I felt the ATLAS program opened a lot of opportunities in the future; taught me a lot of things that I would not really do and helped me find my physical peak”

For the majority of the students, one of the most beneficial and important aspects of the program had been the learning of “how to do things right” and adding to their repertoire of techniques and activities which they could do with friends or by themselves. Many students, who reported engaging in various out of school sports, felt that the newly acquired skills, techniques, and fitness benefits arising from the program were highly transferable, such that they had gained an additional competitive edge. The particular techniques and activities most often referred to in this context were squats, lunges, and CrossFit.

Another frequently mentioned positive aspect of the ATLAS program had been the sense of achievement gained from the evidential gradual improvement in fitness throughout the duration of the program, with many commenting on the enjoyment they had gained from the regular testing of their performance against oneself and their peers;

“At the start of the program in the CrossFit challenges, I was really, really puffed by the end but then at the end of the program I was still getting puffed by them but nowhere near as much and I could run a lot further for a lot longer”

while yet others (albeit a minority) commented favorably on the social aspects of ATLAS;

“… having a training partner, having someone beside you, to slap you across the back of the head and tell you to get up and stop being lazy.”

Engaging in activities which were not usually part of the school curriculum was perceived as a special treat for some students who for instance had been doing boxing as part of their program. Not only did this present a welcome change from normal routine, but also appeared to have aided in feelings of empowerment and improved standing compared to students not involved in ATLAS.

#### Key messages

Students had perceived a range of different key messages being conveyed by the ATLAS program. However, the most frequently mentioned related to the importance of reducing sedentary behavior and in particular screen-time, and reducing the consumption of sugary drinks while increasing water intake. This was closely followed by the importance of staying fit while increasing general physical activity and increasing incidental or opportunistic exercise (e.g., running to the school bus in the morning instead of walking). While many students had taken away several key messages, these four main areas accounted for over 80% of individual comments made.

Other less frequently mentioned key messages were the importance of attending to a healthy diet, with this mostly being associated with reducing overall caloric intake and fat, and the significance of learning and employing the correct technique over sheer strength. Finally, only one student had taken away from the program quite a profound message alluding to the importance of individual capacity and achievement.

“It’s all about going at your own pace and achieving your own goals.”

#### ATLAS app

The reception of the ATLAS smartphone app was somewhat mixed. While approximately 80% of the students participating in the focus groups reported owning a smartphone or iPad, approximately 60% reported having downloaded the ATLAS app. Of those who had not done so, the reasons given were mostly that they had forgotten, were not aware of it, or had neglected to download it due to issues or problems with their device. Of “non-smartphone owners,” only a few students reported having used the Web app. This was exclusively accessed on home PC, and the use of it was in most instances limited to a single viewing.

Of the remaining students, over half reported short-term, once-only or very occasional use of the app, while the remainder (approximately eight students) had used it quite frequently (i.e., three times/week or more). Three students particularly commented that their little usage of the app was due to being unsure how to use it.

In terms of the utility of the specific functions within the app, the My workouts, My steps, and My goals functions were the most frequently used. While the feedback relating to the My workouts function was mostly positive, being described as challenging and enjoyable, a few students had felt it had been boring and repetitive. The My steps function was also a high-use function, but mostly only short-term (limited by accessibility to pedometers), with some reporting simply forgetting about it. However, one student had particularly liked the graphing function to visualize his own performance while, for others, the My steps function had increased their awareness of physical activity or lack thereof. Generally, the use of pedometers was erratic and short-term.

The My goals function had received moderate use and reports were generally good (albeit not very elaborate). Only one student had used my technique function, and had found it very useful. The function which certainly received the most critical feedback was My motivation, which almost exclusively had been considered a nuisance by students. Mostly, the messages had been considered too frequent, too repetitive, or received at inappropriate times (e.g., midnight) and therefore not perceived to fit in with the rhythm of their day. However, one student had found self-programed messages very useful as reminders to engage in physical activity.

The vast majority of students taking part in the focus groups reported having successfully reached the goals which they had set for themselves at the outset of their participation in ATLAS. The most frequently mentioned of these goal accomplishments related to having achieved increased fitness levels including reaching sporting goals;

“My goal was just to get fitter be able to get a place in cross country which I had never done. Well, mostly I’d come 10th. But after the ATLAS program, I actually came 3rd or 4th, so I finally got into zone”

followed by enhanced self-confidence, including higher expectations of oneself, and feelings of mental stamina;

“Before the ATLAS program, I underestimated myself and set my goals pretty low, but during I set them a lot higher and felt I could reach them a lot more easily”

Yet others, who reported having reached their goals, had entered the program with the expectation of becoming more physically active, improving their knowledge of health and nutrition, achieving weight loss, achieving a general improvement in their physical or mental strength, or simply, as one student put it, perform to the “best of [his] ability.”

Of the seven students who reported not having reached their goals, four had not set any specific goals at the outset of the program, while two felt they may have reached their goals had the program continued, while one student, very introspectively, noted that while he had failed to reach his goal of a certain number of steps per day, he had learnt the importance of realistic and progressive goal setting.

#### Diet and sugary drinks

In terms of dietary changes resulting from participation in the ATLAS program, a little over half of the students reported changes to their dietary habits as a direct result of what they had learnt in the ATLAS program. This had taken the form of attempts to purchase and consume healthier foods (less junk foods), reduce amount of food eaten, eating more of the healthier foods served up at home, healthier snacking options and, most commonly, increased fruit intake. There was clear evidence that students had increased their awareness of healthy nutrition, leading to a “think before you buy” approach and a conscious decision to improve long-term health outcomes;

“Yeah it has [changed my attitude to sugary drinks] because after seeing the stuff they showed us about it. It’s just like terrible all the caffeine in like Monsters and all the energy drinks and that, so I’m just sticking with water now.”

Of the remainder of the students, who did not perceive any changes to their diets, most reported already adhering to healthy balanced diets, containing little junk foods, which not surprisingly appeared to stem from healthy family dietary routines. A similar pattern was observed in the students’ attitude toward sugary drinks. While five students reported no changes to their consumption of sugary drinks, three of these indicated that such drinks had not been part of their diet in the first place. The remainder of students described changes to their knowledge and behavior relating to the consumption of sugary drinks, with most having cut down their consumption, and some having either switched to sugar-free soft drinks, or cut out sugary drinks from their diet altogether and as a result increased their water consumption, with one student commenting on the positive effects it has had on his energy levels;

“I like to drink water and [it has] sort of helped me keep going outside, whereas before I [would] get tired and go inside and sit down but now, because I don’t drink as much soft drink and that, I can sort of stay outside for a bit longer”

#### Physical activity

The discussions around the students’ attitude and behaviors relating to physical activity provided strong evidence of the profound impact that the program had exerted, not only on actual behavior, but also on the cognitions accompanying the choices which the students make. The vast majority reported having replaced sedentary behaviors (mainly some form of screen-time) with physically active behaviors such as outside play or intentional fitness activities such as jogging, bike rides, chin-up’s, push-up’s, etc. In many instances, the students talked about these changes having occurred in personal realms as well as social contexts, such that the increased physical activity had extended to friendship circles and family members as well;

“I used practically every afternoon be on my Xbox just playing video games and when the ATLAS program started like now I like practically out every afternoon shooting hoops with my brother and all of that, doing sport and kicking a ball and everything.”

Not surprisingly, there was strong evidence that increased motivation was one of the key factors behind their behavior changes. This motivation appeared to stem from greater enjoyment in physical activity, a more serious approach to fitness, as well as greater knowledge and skill. Indeed, quite a few students talked about the ATLAS program having equipped them with skills and techniques to improve their sporting performance and stamina;

“… with surfing it’s helped, like I got heaps more arm strength now or paddling power, like I’d paddle for like 2 h and I’d be tired. Now I can paddle like the entire day and I’m fine.”

Another of the more profound impacts of the ATLAS program were changes to students’ routines. While some had made just minor changes to everyday routines, such as running to school bus instead of walking, some had adopted an impressive daily fitness routine;

“Well, every time I used to walk home and I used to go for a jog but now I do a little bit more extra; like I’d go for a jog and come home and we’ve got this tree out the back and its kind of like a chin up pole and I’d do chin ups and before I’d go to bed, I do about 50 sit ups.”

### Barriers and challenges to the development, implementation, and evaluation of the ATLAS app

As described previously, the ATLAS app was developed to support the delivery of a school-based obesity prevention program for adolescent boys. However, due to the timing of school terms and the conditions of funding, we were unable to conduct a usability study of the ATLAS app prior to the RCT. Consequently, the app was launched and was made available to participants before we were able to rectify minor technical glitches. More specifically, in the original version of the app, the tailored motivational messaging function did not send the push prompts to the participants’ phones. The function was subsequently fixed by the technical team and participants were encouraged to update their app to access this feature, but this did not occur until 5-weeks into the intervention period.

**Figure 4:**
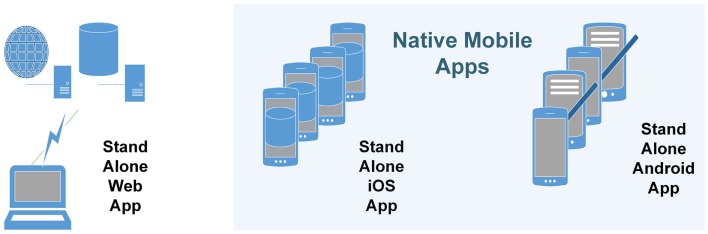
**Original ATLAS infrastructure architecture**.

The diversity of the ATLAS platform and device servicing (i.e., iOS, Android, and web) was designed to provide greater access for users, but came at the cost of higher maintenance and data dispersion issues (Figure 4). The web-based application was primarily written in *Hypertext Preprocessor* and hosted in a data center in India. It included a distributed architecture model traditionally used in standard *n*-tier web applications, in that the client layer, business, and data access layer, and data layer all reside in different locations. Platform decisions were solely based on the two most dominant market shares at the time of development. As shown in Figure 4, each platform has it merits and drawbacks, but both use local device storage; the iOS version can also be backed up to a secondary location such as iTunes or the Cloud. This created problems for the evaluation of the ATLAS app as we did not have access to user data to determine the utility and usage of the five different functions (i.e., *My steps, My workouts, My technique, My goals*, and *My motivation*).

## Discussion

The primary objective was to describe the development and implementation of the ATLAS smartphone app designed to promote physical activity and reduce screen-time in adolescent boys attending schools in low-income communities. Although we were unable to collect objective usage data, the majority of participants reported having access to a smartphone device and utilized the ATLAS app functions to some extent. The focus group findings indicated that the participants benefited from the ATLAS program in general, but did not engage extensively with the smartphone app. Lack of engagement may in part be due to the technical glitches experienced by the research team.

To our knowledge, ATLAS is the first smartphone app designed to supplement an obesity prevention program for adolescent boys. The app included five major features, guided by behavioral theory to promote physical activity and reduce sedentary behavior and the consumption of sugary drinks. A unique aspect of the ATLAS smartphone app was that it included push prompt messages (e.g., to set goals), based on information entered by participants. After completing the program, almost half of the participants agreed or strongly agreed that the push prompt messages reminded them to be more active, reduce their screen-time, and drink less sugary drinks. Interestingly, the messaging was considered a nuisance by many students in the focus groups. However, it is possible that messaging still had its desired effect, as participants’ intentions to adhere to the ATLAS behavioral messages were high at the completion of the study. The content of the push prompt messages used in the ATLAS intervention was guided by the Nutrition and Enjoyable Activity for Teen Girls intervention (11, 12, 37). Similarly, the format and style of messages was guided by formative work conducted with adolescents (36), which found that young people desired SMS that were (i) informative providing relevant new information), (ii) simple (limited to small words and phrases), and (iii) sociable (could be shared easily with friends). The optimal messaging format for promoting health behavior in young people is not known and additional formative research may help to create messages that are meaningful and long-lasting.

Due to the structure of the ATLAS architecture, we were not able to determine the degree to which participants engaged with the app or if they continued to use the app after the completion of the study. It is possible that the novelty of the app wore off quickly, as participants may have found more attractive mobile apps to use. In adults, self-monitoring behaviors diminish over the duration of an intervention (38). While there is evidence to suggest that behavioral skills, such as goal setting and self-monitoring are important for adolescents’ physical activity levels (13, 39–41), evidence for their sustained impact is limited. It is plausible to suggest that adolescents also find it challenging to adhere to physical activity self-monitoring protocols. Few studies have explored the ways that young people use apps to monitor their health behaviors and a number of questions have emerged from our findings. What are the apps most commonly used by adolescents? What are the features of these apps? How many apps do young people have on their phones? Do push prompts encourage young people to engage with specific apps? What other strategies can be used to enhance goal setting and self-monitoring in adolescents? Young people have notoriously short attention spans (42, 43) and can be a challenging group to keep engaged. “Gamification” or “social media linkage” might provide some entertainment value and encourage prolonged use of the app in future versions. These questions could be explored in future research including analyses examining app usage and its association with behavior change along with qualitative research to explore adolescent perceptions and beliefs around these issues.

In the current study, participants were provided with pedometers to self-monitor their physical activity. A recent systematic review concluded that pedometers could be used to increase physical activity in young people and highlighted the importance of individually tailored goals (44). Although these strategies were employed in the ATLAS intervention, only 30% of participants wore their pedometers regularly. In a previous study, Scott and colleagues (45) found that many young people did not enjoy wearing objective monitoring devices such as pedometers and accelerometers and it is possible that participants in the current study were reluctant to wear the devices for self-monitoring purposes. While not feasible in the current study, apps that take advantage of a phone’s inbuilt accelerometer (e.g., “Moves”) may have more utility for promoting self-monitoring in young people. These apps do not require the user to wear an additional activity monitor or regularly enter values, but the user must carry their phone, which can also be considered a limitation. Furthermore, evidence for the validity and reliability of smartphone apps to measure physical activity is only starting to emerge in the literature and acceleration values from phones may not always provide a reliable estimate of a person’s physical activity.

**Figure 5:**
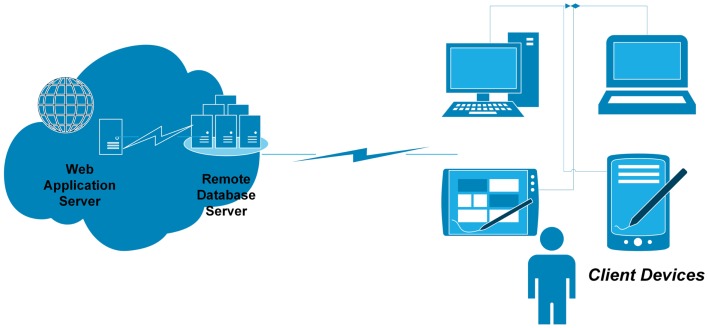
**Proposed ATLAS data storage arrangements**.

Over two-thirds of participants in the intervention group reported access to a smartphone or tablet. Although access is not equivalent to ownership, there is a growing trend in the ownership of smartphone devices in youth populations (15). To cater for participants who did not have access to a smartphone, a website was developed. While the initial multi-versions of ATLAS were suitable for testing and evaluation in the intervention, the challenges of maintaining all three versions for use in future studies and for dissemination to schools was not considered to be feasible. Therefore, at the completion of the study it was decided that the ATLAS app would evolve into a single web services-based architecture. The research team is currently working on a revised ATLAS app including native mobile applications written for both iOS and Android devices. This model will allow the ATLAS app to be serviced on multiple mobile devices and computing platforms due to it becoming effectively a web-based mobile application. Through the use of custom-developed web services, coupled with a centralized data source (Figure 5) we will have significantly more control and access to the application data for research and analysis purposes. With the new architecture, users of all types of smartphones, including Apple, Android, Windows, and Blackberry will be able to consume the service.

Active Teen Leaders Avoiding Screen-time is the first obesity prevention program targeting adolescent boys to be supplemented with a purpose built smartphone app. Despite these strengths, there are some limitations to the current study. First, it was not possible to determine the unique contribution of the app to behavior change, as it was one component of a multi-component school-based intervention. Second, due to the original software architecture we were not able to access participants’ usage data. Alternatively, participants self-reported their use of the app features, which introduces self-report bias. Third, our study was conducted in a sample of adolescent boys attending schools in low-income communities and therefore our findings cannot be generalized to other populations. Finally, the timing of the school-based intervention prevented us from conducting a usability study before implementation and minor technical problems were experienced.

## Conclusion

In this study, we have described the development and implementation of a smartphone app designed to supplement a school-based obesity prevention program for adolescent boys. The majority of participants reported having access to a smartphone or tablet and many engaged with the ATLAS app features. Participants reported moderate satisfaction with the app, but were more positive of the intervention in general. Findings from our focus groups suggest that additional training on how to use the app may be necessary to improve usage in future studies. In addition, the technical glitches experienced by the research team highlight the importance of allowing sufficient time to conduct a usability study before conducting a full-scale RCT.

Although eHealth interventions hold promise for behavior change in youth, it is unlikely that they will provide the “silver bullet” to the global physical activity pandemic. Physical activity is a complex behavior that can take place in a wide variety of settings and is influenced by various psychological, social, and environmental factors. Future studies are encouraged to explore the utility of technology-based intervention strategies, such as smartphone apps, to determine if they are appropriate stand-alone strategies or adjuncts to face-to-face behavior change interventions.

## Author Contributions

David R. Lubans and Philip J. Morgan obtained funding for the research. All authors contributed to developing the protocols and reviewing, editing, and approving the final version of the paper. David R. Lubans, Philip J. Morgan, and Jordan J. Smith developed the intervention materials. All authors have read and approved the final manuscript.

## Conflict of Interest Statement

The authors declare that the research was conducted in the absence of any commercial or financial relationships that could be construed as a potential conflict of interest.
